# The Effect of Polarized Training Intensity Distribution on Maximal Oxygen Uptake and Work Economy Among Endurance Athletes: A Systematic Review

**DOI:** 10.3390/sports12120326

**Published:** 2024-11-27

**Authors:** Henrik Lyngstad Nøst, Morten Andreas Aune, Roland van den Tillaar

**Affiliations:** Department of Sport Sciences and Physical Education, Nord University, 7600 Levanger, Norway; henrik.l.nost@student.nord.no (H.L.N.); morten.a.aune@nord.no (M.A.A.)

**Keywords:** high-intensity training, low-intensity training, HIT, LIT, VO_2max_

## Abstract

High-intensity training (HIT) has commonly been the most effective training method for improvement in maximal oxygen uptake (VO_2max_) and work economy, alongside a substantial volume of low-intensity training (LIT). The polarized training model combines both low- and high-intensity training into a specific training intensity distribution and has gained attention as a comprehensive approach. The objective of this review was to systematically search the literature in order to identify the effects of polarized training intensity distribution on VO_2max_, peak oxygen uptake (VO_2peak_), and work economy among endurance athletes. A literature search was performed using PubMed and SPORTDiscus. A total of 1836 articles were identified, and, after the selection process, 14 relevant studies were included in this review. The findings indicate that a polarized training approach seems to be effective for enhancing VO_2max_, VO_2peak_, and work economy over a short-term period for endurance athletes. Specifically, a training intensity distribution involving a moderate to high volume of HIT (15–20%) combined with a substantial volume of LIT (75–80%) appears to be the most beneficial for these improvements. It was concluded that polarized training is a beneficial approach for enhancing VO_2max_, VO_2peak_, and work economy in endurance athletes. However, the limited number of studies restricts the generalizability of these findings.

## 1. Introduction

Endurance training, which leads to positive results, always involves adjustments of the duration, intensity, and frequency of training sessions [1,2]. Therefore, sports that require endurance always consist of planning and adjustments of endurance training [3,4,5], aiming to meet the crucial work demands required to perform. Furthermore, the relative effects of various combinations of duration and training intensity distribution have been a subject of study and debate for decades among athletes, coaches, and scientists [2].

To gain deeper insights, three common approaches to training intensity are used in endurance training aiming at enhancing performance [2,6]. Low-intensity training (LIT) is characterized by speeds adjusted for longer durations, often referred to as long slow distance training below the first ventilatory threshold, or <2 mmol·L^−1^. Moderate-intensity training (MIT) involves intensities commonly performed continuously or in intervals between the two ventilatory thresholds, typically ranging from 2 to 4 mmol·L^−1^. While high-intensity training (HIT), corresponds to speeds above the second ventilatory threshold, or >4 mmol·L^−1^, and is primarily conducted as interval training, intermittent intervals, or short, high-intensity sprints [2,7].

High-intensity training has commonly been the most effective training approach for improvements in endurance sports performance [8]. This includes improvements in crucial performance factors such as maximum oxygen uptake (VO_2max_) and work economy among endurance athletes [3,9]. VO_2max_ represents the highest rate at which an individual can consume oxygen (O_2_) during intense exercise, reflecting aerobic capacity and cardiovascular fitness [10]. Enhanced VO_2max_ supports better O_2_ delivery and energy production, making it a key performance indicator in endurance sports [3,5,10]. Work economy, on the other hand, indicates how efficiently an athlete uses O_2_ during at a given intensity [11]. An improved work economy means that less O_2_ is needed to maintain a given intensity, conserving energy and delaying fatigue [12]. This efficiency is crucial for resisting fatigue and enhancing performance, especially in long-distance competitions [3,5,11].

However, an approach consisting of a high volume of HIT can thus lead to undesirable effects that can cause inadequate recovery, which is also related to a decrease in performance [13,14]. Therefore, substantial volumes of LIT appear to be a crucial component of endurance training, as it may create a foundation for the specific adaptations that result from HIT [6,15]. Additionally, LIT has also shown a great ability to increase recovery from HIT [16], and a combination of both LIT and HIT has, therefore, been mentioned in previous papers as optimal for the improvement of endurance performance [2,6]. At the same time, it is still unclear how the distribution of LIT and HIT should be modeled.

Traditionally, endurance athletes follow several common training programs, such as the threshold model, typically emphasizing > 40% MIT, and the pyramidal model, emphasizing a high volume of LIT (>70%), with progressively smaller proportions of MIT and HIT [2,7]. Another widely used approach, the polarized training model, uniquely combines low- and high-intensity into a specific intensity distribution. This model primarily consists of a high volume of LIT, with a smaller but substantial amount of HIT, and a relatively small proportion of MIT. As a result, this leads to an intensity distribution consisting of approximately 70–75% volume LIT, 0–5% MIT, and 15–20% HIT [2,7,17]. Therefore, the polarized training model is characterized by specific high-intensity sessions separated by several low-intensity workouts, with the training intensity being tightly monitored [18].

It has been discussed whether polarized training is optimal or not for endurance athletes [17,19]. Seiler [2] mentioned a typical training intensity distribution consisting of 80% LIT and 20% high-intensity work of competitive endurance athletes. Additionally, Laursen [6] suggested polarized training where ~75% of the total training volume is performed at low intensities and 10–15% performed at high intensities as an optimal training intensity distribution for elite athletes performing at endurance competitions. This also corresponds well with another study, where a polarized training distribution consisting of ~73% LIT and ~14% HIT significantly improved 10 km running performance among recreational athletes [20]. Therefore, it was concluded that polarized training could stimulate great performance-related effects after a 10-week intervention period. Moreover, a previous review comparing polarized training with threshold training suggested that polarized training resulted in a significantly greater improvement in time-trial performance compared to threshold training [21]. This also underlines the potential benefits of polarized training for performance improvement.

Based on this, it can be assumed that similar significant improvements may also impact other parameters related to endurance performance. The previous literature has mentioned VO_2max_ and work economy as two crucial factors determining the ability to perform in endurance sports [3,5,10]. However, current reviews still lack some knowledge regarding the specific effects of polarized training on VO_2max_ and work economy across a range of endurance sports [21,22]. Although both systematic reviews and meta-analyses provide research summaries, a systematic review was conducted due to the limited number of studies that directly compare the effects of polarized training. Therefore, this present systematic review aims to examine whether polarized training intensity distribution positively affects maximal oxygen uptake and work economy among endurance athletes.

## 2. Materials and Methods

### 2.1. Literature Search

This systematic review was conducted following the 2020 guidelines and checklists established by the Preferred Reporting Items for Systematic Reviews and Meta-Analyses (PRISMA) (Table S1) [23]. The used keywords in this literature search were combined with Boolean operators (AND, OR) in the research process. No limitations were placed on publication dates due to the relatively limited number of studies published on this subject. A SPORTDiscus and PubMed literature search from inception to 14th of April 2024 was performed. The title, abstract, and keywords were searched using the following search strategy: ((“polarized” OR “POL” AND “training method”) AND (“endurance” OR “aerobic” AND “sports” OR “exercises” OR “activities”)).

### 2.2. Inclusion Criteria

**Figure 1:**
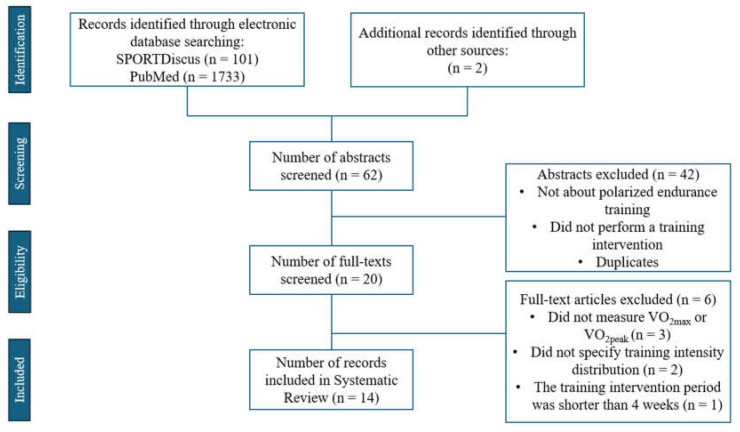
Flow chart of the search strategy and study selection.

Studies were included in this review if the following inclusion criteria were met: (1) They analyzed the effects of polarized endurance training on VO_2max_ or VO_2epak_ and/or measures of work economy. (2) They involved at least a 4-week training intervention, and employed a one-, two, or multiple-group- crossover design. (3) The participants were at least at a recreational level or higher. (4) The studies included were in English and published in a peer-reviewed journal. Due to the limited studies published on the effects of polarized training on endurance sports, gender and training background were not considered as differentiating factors. A flowchart of the literature search strategy and study selection process is presented in Figure 1.

### 2.3. Data Extraction

The results from the database search were imported into the software for bibliographic management (Endnote version 21 for Windows). The abstracts were evaluated in this software, and, if the abstracts exhibited potential, the full texts of the articles were read. The articles were then examined for whether or not they met the inclusion criteria.

The studies included had to measure baseline-, post-, and change-values for at least one following variables: work economy during a given submaximal intensity (velocity/power at 4 mmol·L^−1^, anaerobic threshold, second lactate threshold, or second ventilatory threshold), and/or maximal oxygen uptake (ml·min^−1^·kg^−1^), and/or reported as either VO_2max_ or VO_2peak_. VO_2max_ refers to the plateau of O_2_ consumption reached, while VO_2peak_ indicates the highest O_2_ consumption attained during an incremental test [24].

Furthermore, the following characteristics from each study were also extracted: authors with publication year, sample size, training status of the participants, the duration of the training interventions, and training intensity distribution.

### 2.4. Quality Assessment

The methodology quality and risk of bias for each study were evaluated by two independent observers using the PEDro scale [25] (Table 1). The PEDro scale includes 11 items to evaluate scientific rigor. Item 1 assesses external validity (yes/no), while items 2–11 rate internal validity, where each is scored as 0 (absent) or 1 (present), giving a total score out of 10. Due to the impracticality of blinding assessors, and the inability to blind the participants and investigators in supervised exercise interventions, items 5–7 related to blinding were removed from the scale [26], following the approach of previous systematic reviews [25]. Consequently, the highest total score was 7 instead of 10. Studies with quality scores ranging from 6 to 7 were classified as “excellent”, a score of 5 was considered “good”, a score of 4 was rated as “moderate”, and studies that scored 0–3 were considered to be “poor” [25].

## 3. Results

### 3.1. Study Selection

A literature search on the databases SPORTDiscus and PubMed identified 1836 potentially relevant articles (Figure 1). A total of 7 duplicates were removed, and 20 articles were selected for full-text review after screening of the titles and abstracts. After the screening of the full texts, 6 records were eliminated with the following reasons: (1) the studies did not measure either VO_2max_, VO_2peak_, or work economy (n = 3); (2) the excluded studies did not specify training intensity distribution or did not match a training intensity distribution primarily consisting of LIT and HIT, which characterizes the polarized training method (n = 2); (3) the training intervention period was shorter than 4 weeks (n = 1). After the selection, a total of fourteen studies met the inclusion criteria and were included in this systematic review.

### 3.2. Quality of Studies

Regarding the quality of the selected studies in this review, the mean score on the PEDro scale was 4.5 ± 0.65, with values ranging from 3 to 5 (Table 1). Eight studies achieved a rating of good quality, five studies were of moderate quality, while the remaining one study was classified as poor quality (Table 1).

### 3.3. Characteristics of Participants

Four studies performed polarized training interventions on recreational participants, six included well-trained participants, and four studies were conducted on elite athletes (elite junior, national elite, and national team). A total of 163 participants (129 men and 34 women) were included in the selected studies, with an age ranging from 17 to 44 years.

### 3.4. Characteristics of the Included Studies

The characteristics of the selected studies are presented in Table 2. Of the included studies, four performed polarized training interventions in running [31,32,38,41], two studies were performed on triathletes [36,37], two in swimming [27,28], one on rowers [39], one on mountain bikers [40], and one study in cycling [34]. Three studies also included more than one endurance sport in their study design. Two of these studies included running, cycling, triathlon, and cross-country skiing [29,35], while another study performed polarized training intervention on both cross-country skiers and biathletes [30].

Two of the included studies had a four-group-comparison design, one study had a three-group design, ten had a two group-design, and one study had a one-group design. The mean length of the training interventions was 9.1 ± 2.9 weeks, with a duration ranging from 4 to 13 weeks.

Of the fourteen studies included, eight measured baseline, post-, and change-values in VO_2max_ or VO_2peak_ and work economy, four studies only measured work economy, while two studies measured only VO_2max_ after the polarized training intervention.

### 3.5. Training Intensity Distribution

The average training intensity distribution across the eleven included studies was 81.3 ± 8.0% LIT, 3.4 ± 3.2% MIT, and 15.4 ± 6.3% HIT. Treff, et al. [39] and Röhrken, et al. [37] showed the largest volume of LIT (respectively, 92 and 91%), while the lowest percentage of LIT (68%) was observed in two studies [29,35]. Additionally, four studies reported 0% as their total training volume of MIT [28,34,37,40], with the largest volume of MIT (11%) found in Carnes and Mahoney [31]. Lastly, Stöggl and Sperlich [29] and Stöggl and Björklund [35] reported the highest training volume of HIT (26%) among the included studies, while Kim, et al. [30] and Treff, et al. [39] had the smallest HIT volume (7% and 6%, respectively) in their polarized training interventions.

### 3.6. Maximal Oxygen Uptake

Improvements in VO_2max_ or VO_2peak_ were found in eight of the ten studies that measured these variables. Jaime Arroyo-Toledo, et al. [28] demonstrated a significant increase in VO_2max_ (+29.9%, *p* = 0.01, Cohen’s d = 0.16) after a 12-week training period. Similarly, Carnes and Mahoney [31] reported a significant increase in VO_2max_ (+8.5%, *p* ≤ 0.05, Cohen’s d = 0.85) after a 12-week intervention. In addition, Stöggl and Sperlich [29] found a significant improvement in VO_2peak_ (+11.7%, *p* < 0.001) following nine weeks of training intervention, while Filipas, et al. [33] also reported a significant increase in VO_2peak_ (+2.1%, *p* < 0.05, Cohen’s d = 0.40) after eight weeks. Kim, et al. [30] observed a 7.0% (*p* > 0.05) increase in VO_2max_ after a 12-week training period, and Selles-Perez, et al. [36] reported a significant improvement in VO_2max_ in cycling (+6.5%, *p* = 0.027, Cohen’s d = 0.95), but not in running (+5.1%, *p* = 0.072, Cohen’s d = 0.69), after 13 weeks. Furthermore, Festa, et al. [32] documented a small increase in VO_2max_ (+1.2%, *p* > 0.05, Cohen’s d = 0.10) following eight weeks of polarized training. Finally, Treff, et al. [39] found a possible small increase in VO_2max_ (+0.6, *p* = 0.67, Cohen’s d = 0.00) after a nine-week intervention. In contrast, Pla, et al. [27] reported a possible small negative effect on VO_2max_ (−0.9%, *p* > 0.05) after a six-week training program, which was similar to the small decrease (−1.6%, *p* = 0.346, Cohen’ d = 0.17) observed by Pérez, et al. [38] following twelve weeks of polarized training.

### 3.7. Work Economy

Ten studies reported data on improvements in work economy. Festa, et al. [32] reported significant improvement in running economy (+5.3%, *p* = 0.04, Cohen’s d = 0.40). Similarly, Filipas, et al. [33] also observed a significant improvement in work economy (+2.1%, *p* < 0.001, Cohen’s d = 0.25), where it was measured as velocity at 4 mmol·L^−1^. Following a four-week intervention, a significant increase of +6.1% (*p* = 0.022) was reported by Schneeweiss, et al. [40]. Furthermore, Stöggl and Sperlich [29] also reported a significant improvement in velocity/power at 4 mmol·L^−1^ by 8.1% (*p* < 0.01). Similarly, Selles-Perez, et al. [36] reported a significant improvement in the second ventilatory threshold in cycling (+7.5%, *p* = 0.043, Cohen’s d = 0.58) and an improvement in running VT_2_ (+2.6%, *p* = 0.102, Cohen’s d = 0.61). Also, Kim, et al. [30] noted an 8.3% (*p* = 0.499) improvement in performance at the anaerobic threshold, while Neal, et al. [34] observed a similar increase in the second lactate threshold (+8%, *p* > 0.05, Cohen’s d = 0.57) after six weeks of polarized training, but these were all not significant. Furthermore, Röhrken, et al. [37] observed non-significant increases in performance at the second lactate threshold in both running (+4.2%, *p* > 0.05) and cycling (+4.5%, *p* > 0.05) following six weeks of training intervention. In addition, Pérez, et al. [38] reported a small improvement in the second ventilatory threshold (+1%, *p* = 0.476, Cohen’s d = 0.11), while Pla, et al. [27] found a possible trivial increase (+0.9%, *p* > 0.05) in velocity at 4 mmol·L^−1^. Finally, two studies reported a small decrease in work economy. Stöggl and Björklund [35] reported a small decrease in velocity/power at 4 mmol·L^−1^ (−1.6%, *p* > 0.05) following nine weeks of training intervention, while Treff, et al. [39] found a small decrease in power output at 4 mmol·L^−1^ (−0.5%, *p* = 0.770, Cohen’s d = −0.05).

## 4. Discussion

The main aim of the present systematic review was to investigate the effects of polarized training intensity distribution on VO_2max_, VO_2peak_, and work economy among endurance athletes. The key findings were that polarized endurance training provides positive effects for VO_2max_, VO_2peak_, and work economy. Improvements in VO_2max_ or VO_2peak_ were found in eight of the ten studies that measured this variable. Among the eight studies reporting improvements in VO_2max_ or VO_2peak_, five demonstrated a statistically significant increase [28,29,31,33,36]. Moreover, improvements in work economy were found across ten of the included studies, with five reporting a significant increase [29,32,33,36,40].

### 4.1. Maximal Oxygen Uptake and Work Economy

The results indicated that eight studies demonstrated improvements in VO_2max_ and VO_2peak_ following polarized training interventions, while five of these were significant. These improvements were found in several endurance sports, including swimming, running, triathlon, cross-country skiing, biathlon, and cycling. Notably, enhancements in VO_2max_ and VO_2peak_ attributed to polarized training also seem to occur regardless of the level of the participants, with significant improvements in VO_2max_ and VO_2peak_ found among recreational, well-trained, and elite endurance athletes.

The effect sizes for VO_2max_ and VO_2peak_ varied from trivial to large, according to Hopkins, et al. [42]. Interestingly, Jaime Arroyo-Toledo, et al. [28] reported a trivial effect size (Cohen’s d = 16), meaning that the actual benefit to performance in swimming might be limited in practice, despite a significant increase in VO_2max_ (+29.9%, *p* = 0.01). In contrast, Selles-Perez, et al. [36] reported a large effect size (Cohen’s d = 0.95) and a significant increase in VO_2max_ (+6.5%, *p* ≤ 0.027), while Carnes and Mahoney [31] reported similar findings (+8.5%, *p* ≤ 0.05, Cohen’s d = 0.85). These findings indicate more practical improvements that are likely to positively impact performance in running and cycling.

Another interesting finding in this review is that most studies reported larger improvements in VO_2max_ and VO_2peak_ utilized when using a higher volume of HIT (18–26%). This could suggest that higher volumes of HIT may play a critical role in enhancing both VO_2max_ and VO_2peak_. For example, Treff, et al. [39] did not find any changes in VO_2max_ (+0.6%, *p* = 0.67), possibly due to the relatively low volume of HIT (6%). Moreover, this also underpins the findings in Talsnes, et al. [43], where an increased load of HIT elicited better VO_2max_ adaptations. Based on the findings in this review, a HIT volume (18–26%) combined with a substantial volume of LIT (70–80%) may be the most beneficial for improvement in VO_2max_ and VO_2peak_.

On the other hand, two studies [27,38] reported a possible decrease in VO_2max_ (−1.6% and −0.9%, respectively). The duration of the study by Pla, et al. [27] was relatively short (only six weeks), which contrasts with studies that have investigated more significant increases in VO_2max_ or VO_2peak_. Simultaneously, it has also been observed that enhancements in VO_2max_ are typically more prominent among young to middle-aged adults [44], which could explain the trivial decrease found by Pérez, et al. [38].

Significant improvements in work economy were observed in five studies [29,32,33,36,40]. However, these studies reported only small to moderate effect sizes. Hence, Filipas, et al. [33] reported the smallest effect size (Cohen’s d = 0.25), while Selles-Perez, et al. [36] was the only study to demonstrate both a significant improvement and a moderate effect size (Cohen’s d = 0.61). This indicates that the interventions likely had a meaningful impact on the work economy, but the practical impact of some of the interventions may be limited. Despite that, polarized training has nevertheless been shown to significantly improve 10 km running performance [20]. Based on the findings in this review, a polarized training intensity distribution emphasizing a substantial volume of LIT (80–90%), combined with a moderate to high proportion of HIT (10–20%), appears to be optimal for enhancing the work economy.

A training intensity distribution consisting predominantly of LIT combined with HIT sessions allows athletes to repeatedly practice the movement patterns specific to their sport. The high volume of LIT in polarized training enables athletes to practice the movement patterns for long durations without excessive fatigue [43], allowing for more repetitions of the specific skill. Meanwhile, the HIT sessions provide opportunities for athletes to practice more repetitions at speeds that occur in competitions, leading to more economical movement patterns [45]. This aligns with the specificity principle, which has been shown over decades to be important in skill development [46].

### 4.2. Physiological Adaptations of HIT and LIT

The polarized training model combines a high volume of LIT with a smaller, but substantial, proportion of HIT into a specific intensity distribution [2,7,17]. Across the studies reviewed, the volume of HIT ranged from 7% to 26% of the total training volume, with findings suggesting that the most optimal outcomes in terms of VO_2max_ and VO_2peak_ may seem to occur in the 17% to 26% range of HIT volume. This is crucial in the polarized training model, as HIT sessions have shown a great ability to stimulate central adaptations in the cardiovascular system, such as increased cardiac contractility, increased stroke volume, and minute volume, which all are associated with enhancements in VO_2max_ [6,9,47].

However, it is important to note that HIT sessions alone, especially at high volumes, which typically occur in the polarized training model, may not be sufficient to sustain long-term improvements. This could lead to undesirable effects that can cause inadequate recovery, which is also related to decreased performance [13,14]. In this context, a substantial volume of LIT in the polarized training model would be important to balance the total training load, allowing for more consistent training without excessive fatigue. In other words, it seems that LIT sessions may be crucial, as they provide a platform for specific adaptations to HIT sessions [15].

Simultaneously, the total training time of LIT has indeed shown positive associations with peripheral adaptations, such as increased mitochondrial density, capillary density, and the development of Type I muscle fibers [6,47,48]. These adaptations are also associated with improvements in VO_2max_ and work economy, leading to enhanced endurance performance [2,7]. Therefore, to ensure balance in the stimulus use of both central and peripheral adaptations, combining specific HIT sessions separated by several LIT workouts into a polarized training intensity distribution seems likely to produce positive benefits in performance factors such as VO_2max_ and work economy.

### 4.3. Further Research and Limitations

It is crucial to note that this systematic review has certain limitations that should be considered when interpreting the results. The sample size was relatively small, due to the limited number of studies published on this subject. This is especially noteworthy for some of the included studies, where a small sample size can potentially impact the results. It may also limit the generalizability of the findings. Another limitation of this systematic review is the presence of publication bias. Studies that do not demonstrate increases in their results are often more difficult to publish.

Furthermore, this review only investigated the short-term effects of the interventions, which means that it remains ambiguous whether similar improvements would be sustained over a more extended period [49]. However, positive changes in endurance performance following a long-term polarized training program were observed. Nonetheless, more research examining the long-term impacts of polarized training is needed.

### 4.4. Practical Applications

A polarized pattern emerges as an effective training intensity distribution approach for endurance athletes. The findings in the present review suggest that coaches and athletes should consider a polarized training model in their training programs. Specifically, a distribution consisting of a moderate to high proportion of HIT volume (17–26%) combined with a substantial volume of LIT (75–85%) may be the most beneficial for improvement in VO_2max_, VO_2peak_, and work economy. Since there is still a lack of research regarding the long-term effects of polarized training, it could be recommended to implement blocks of polarized training in the ordinary training program. This can be beneficial as part of a tapering process or during periods requiring effective adaptations to VO_2max_, VO_2peak_, and work economy.

## 5. Conclusions

In conclusion, this systematic review aimed to investigate the effects of polarized training intensity distribution on VO_2max_, VO_2peak_, and work economy among endurance athletes. The results from the selected studies suggest that polarized training is an effective approach for improvement in VO_2max_, VO_2peak_, and work economy. However, given the observed effect sizes ranging from trivial to large, there are still some concerns about the practical impact of polarized training. Additionally, it should be noted that it remains ambiguous whether similar improvements would be sustained over a more extended period, due to a lack of research. Consequently, more research examining both the practical and long-term impacts of polarized training is needed.

## Figures and Tables

**Table 1:** PEDro ratings of the included studies.

Study	PEDro Scale: Item Number									
	1	2	3	4	5	6	7	8	Total Score	Rating
Pla, et al. [27]	Yes	1	0	1	0	0	1	1	4	Moderate
Jaime Arroyo-Toledo, et al. [28]	Yes	0	0	1	1	0	1	1	4	Moderate
Stöggl and Sperlich [29]	Yes	1	0	1	1	0	1	1	5	Good
Kim, et al. [30]	Yes	0	0	1	0	0	1	1	3	Poor
Carnes and Mahoney [31]	Yes	1	0	1	1	0	1	1	5	Good
Festa, et al. [32]	Yes	1	0	1	1	0	1	1	5	Good
Filipas, et al. [33]	Yes	1	0	1	1	0	1	1	5	Good
Neal, et al. [34]	Yes	1	0	1	1	0	1	1	5	Good
Stöggl and Björklund [35]	Yes	1	0	1	1	0	1	1	5	Good
Selles-Perez, et al. [36]	Yes	1	0	1	0	0	1	1	4	Moderate
Röhrken, et al. [37]	Yes	1	0	1	0	0	1	1	4	Moderate
Pérez, et al. [38]	Yes	1	0	1	1	0	1	1	5	Good
Treff, et al. [39]	Yes	1	0	1	1	0	1	1	5	Good
Schneeweiss, et al. [40]	Yes	1	0	1	0	0	1	1	4	Moderate

Items in the PEDro scale: 1 = eligibility criteria were specified; 2 = subjects were randomly allocated to groups; 3 = allocation was concealed; 4 = the groups were similar at baseline regarding the most important prognostic indicators; 5 = measures of 1 key outcome were obtained from 85% of subjects initially allocated to groups; 6 = all subjects for whom outcome measures were available received the treatment or control condition as allocated or, where this was not the case, data for at least 1 key outcome were analyzed by “intention to treat”; 7 = the results of between-group statistical comparison are reported for at least 1 key outcome; 8 = the study provides both point measures and measures of variability for at least 1 key outcome.

**Table 2:** Characteristics of the studies and participants.

		Participants			Training Characteristics			
Study	Sport	n (Men/Women) Age (Years)	Level	VO_2max_ Baseline (mL·min^−1^·kg^−1^)	Duration (Weeks)	Intensity Distribution % (LIT-MIT-HIT)	Measures	Results
Pla, et al. [27]	Swimming	22 (12/10) 17 ± 3.0	Elite junior	56.0 ± 11.3	6	81-4-15	VO_2max_ (mL·min^−1^·kg^−1^) V_4_ (m·s^−1^)	Possibly small ↓ on VO_2max_ (−0.9%, *p* > 0.05) Possibly small ↑ on V_4_ (+0.9%, *p* > 0.05)
Jaime Arroyo-Toledo, et al. [28]	Swimming	6 (0/6) Adolescents	National elite	30.45 ± 6.6	12	82-0-18	VO_2max_ (mL·min^−1^·kg^−1^)	Significant ↑ of VO_2max_ (+29.9%, *p* = 0.01)
Stöggl and Sperlich [29]	Running, cycling, triathlon, and cross-country skiing	12 (12/0) 31 ± 6.0	Well- trained	60.6 ± 8.3	9	68-6-26	VO_2peak_ (L·min^−1^·kg^−1^) LT_4_ (km·h^−1^ or W)	Significant ↑ in VO_2peak_ (+11.7%, *p* < 0.001) Significant ↑ in LT_4_ (+8.1%, *p* < 0.01)
Kim, et al. [30]	Cross-country skiing and biathlon	16 (8/8) 23.4 ± 3.8	National team	71.05 ± 7.90	12	91-2-7	VO_2max_ (mL·min^−1^·kg^−1^) AT (L·min^−1^)	VO_2max_ ↑ by +7.02% (*p* = 0.069) Performance at AT ↑ by +8.2% (*p* = 0.499)
Carnes and Mahoney [31]	Running	9 (8/1) 44.2 ± 14.6	Recrea- tional	45.9 ± 7.1	12	74-11-15	VO_2max_ (mL·min^−1^·kg^−1^)	Significant ↑ of VO_2max_ (+8.5%, *p* ≤ 0.05)
Festa, et al. [32]	Running	19 (15/4) 43.2 ± 8.4	Recrea- tional	53.0 ± 5.9	8	77-3-20	VO_2max_ (mL·min^−1^·kg^−1^) RE (ml·kg^−1^·km^−1^)	VO_2max_ ↑ by +1.2% (*p* > 0.05) Significant ↑ in RE (+5.3%, *p* = 0.04)
Filipas, et al. [33]	Running	15 (15/0) 38 ± 5.0	Well- trained	68.0 ± 4.0	8	80-6-14	VO_2peak_ (mL·min^−1^·kg^−1^) LT_4_ (km·h^−1^)	Significant ↑ of VO_2peak_ (+2.1%, *p* < 0.05) Significant ↑ of LT_4_ (+1.2%, *p* < 0.001)
Neal, et al. [34]	Cycling	11 (11/0) 37 ± 6.0	Well- trained	-	6	80-0-20	LT_2_ (W)	LT_2_ ↑ by +9% (*p* > 0.05)
Stöggl and Björklund [35]	Cross-country skiing, cycling, triathlon, and running	12 (11/1) 31 ± 6.0	Well- trained	-	9	68-6-26	LT_4_ (watt or km·h^−1^)	Possibly small ↓ in LT_4_ (−1.6%, *p* > 0.05)
Selles-Perez, et al. [36]	Triathlon	6 (6/0) 28.5 ± 7.7	Recrea- tional	50.5 ± 2.9 (bike) 52.8 ± 4.1 (run)	13	85-4-11	Bike and run VO_2max_ (mL·min^−1^·kg^−1^) Bike VT_2_ (W) Run VT_2_ (m·s^−1^)	Bike VO_2max_ significant ↑ by +6.5% (*p* = 0.027) Run VO_2max_ ↑ by +5.1% (*p* = 0.072) Bike VT_2_ significant ↑ by +7.5% (*p* = 0.043) Run VT_2_ ↑ by +2.6% (*p* = 0.10)
Röhrken, et al. [37]	Triathlon	7 (5/2) 29.1 ± 7.6	Well- trained		6	92-0-8	Run LT_2_ (km·h^−1^) Bike LT_2_ (W)	Run LT_2_ ↑ by +4.2% (*p* > 0.05) Bike LT_2_ ↑ by +4.5% (*p* > 0.05)
Pérez, et al. [38]	Running	11 (11/0) 40.6 ± 9.7	Recrea- tional	55.8 ± 4.9	12	80-4-16	VO_2max_ (mL·min^−1^·kg^−1^) VT_2_ (mL·min^−1^·kg^−1^)	Small ↓ in VO_2max_ by −1.6% (*p* = 0.35) VT_2_ ↑ by +1% (*p* = 0.476)
Treff, et al. [39]	Rowing	7 (7/0) 21 ± 2.0	National elite	68.0 ± 7.0	11	93-1-6	VO_2max_ (mL·min^−1^·kg^−1^) P_4_ (W)	Possibly small ↑ in VO_2max_ (+0.6%, *p* = 0.68) Possibly small ↓ in P_4_ (−0.5%, *p* = 0.77)
Schneeweiss, et al. [40]	Mountain bike	10 (8/2) 18.4 ± 4.7	Well- trained		4	87-0-13	LT_4_ (W)	Significant ↑ in LT_4_ (+6.1%, *p* = 0.02)

AT, anaerobic threshold; HIT, high-intensity training; LIT, low-intensity training; LT_2_, second lactate threshold; LT_4_, lactate threshold at 4 mmol·L^−1^; MIT, moderate-intensity training; P_4_, power output at 4 mmol·L^−1^; RE, running economy; VO2max, maximal oxygen uptake; VO_2peak_, peak oxygen uptake; VT_2_, second ventilatory threshold; V_4_, velocity at 4 mmol·L^−1^; W, watts; Symbols: ↑, increased; ↓, decreased.

## Data Availability

Not applicable.

## Supplementary Materials

The following supporting information can be downloaded at: https://www.mdpi.com/article/10.3390/sports12120326/s1, Table S1: PRISMA 2020 Checklist.
